# Supplementary material for: Coronary heart disease risk prediction based on GAIN imputation and interpretable machine learning
Source: Front Genet. 2026 Jan 21;16:1752811. doi: 10.3389/fgene.2025.1752811 (PMC12867338; doi:10.3389/fgene.2025.1752811)
Supplement: Supplementary file 1 [file Table1.docx]

Supplementary Material

# Supplementary Tables

Table 1 Description of categorical features

| Feature |  | CHD | Non-CHD |
| --- | --- | --- | --- |
| Blood type | A | 5928 | 5190 |
|  | B | 4980 | 4316 |
|  | O | 6929 | 6402 |
|  | AB | 1482 | 1254 |
|  | Missing rate% | 1.82 | 2.23 |
| Gender | male | 12746 | 9262 |
|  | female | 6932 | 8496 |
| alcohol drinking | yes | 7316 | 4079 |
|  | no | 12126 | 13475 |
|  | Missing rate% | 1.20 | 1.15 |
| smoke | yes | 9816 | 4927 |
|  | no | 9626 | 12627 |
|  | Missing rate% | 1.20 | 1.10 |
| diabetes | yes | 5575 | 1912 |
|  | no | 14103 | 15846 |
| hypertension | yes | 9513 | 3980 |
|  | no | 10165 | 13778 |

Table 2 Description of Numerical Features

| Feature | Abbreviation | Unit | CHD_mean | CHD_std | CHD_missing_rate% | non-CHD_mean | non-CHD_std | non-CHD_missing_rate% |
| --- | --- | --- | --- | --- | --- | --- | --- | --- |
| age | age | years | 70.81 | 10.95 | 0 | 60.96 | 14.33 | 0 |
| D-dimer | D-D | mg/L FEU | 0.5 | 0.38 | 17.42 | 0.47 | 0.37 | 20.37 |
| high-sensitivity troponin I | hs-cTnI | µg/L | 0.005 | 0.0045 | 38.88 | 0.003 | 0.0037 | 9.32 |
| hemoglobin | HB | g/L | 120.73 | 21.76 | 0.09 | 122.07 | 21.27 | 0.24 |
| white blood cell count | WBC | *10^9/L | 6.25 | 2.1 | 3.38 | 6.25 | 2.22 | 4.01 |
| lymphocyte percentage | LYM% | % | 22.2 | 9.64 | 0.38 | 23.63 | 10.89 | 0.46 |
| monocyte percentage | MO% | % | 8.22 | 2.63 | 2.09 | 7.84 | 2.64 | 1.4 |
| neutrophil percentage | NE% | % | 66.57 | 11.46 | 0.5 | 65.77 | 12.78 | 0.57 |
| eosinophil percentage | EO% | % | 1.99 | 1.6 | 3.73 | 1.8 | 1.56 | 3.1 |
| basophil percentage | BA% | % | 0.41 | 0.26 | 0.8 | 0.4 | 0.27 | 0.61 |
| neutrophil count | NE | *10^9/L | 4.12 | 1.72 | 4.79 | 4.06 | 1.86 | 5.61 |
| lymphocyte count | LYM | *10^9/L | 1.32 | 0.57 | 0.9 | 1.4 | 0.6 | 1.11 |
| monocyte count | MO | *10^9/L | 0.51 | 0.2 | 2.69 | 0.48 | 0.2 | 2.44 |
| eosinophil count | EO | *10^9/L | 0.11 | 0.09 | 4.76 | 0.1 | 0.09 | 3.73 |
| basophil count | BA | *10^9/L | 0.02 | 0.02 | 2.08 | 0.02 | 0.02 | 1.73 |
| red blood cell count | RB | *10^12/L | 4.01 | 0.72 | 0.21 | 4.08 | 0.71 | 0.43 |
| hemato crit | HCT | -- | 0.37 | 0.06 | 0.16 | 0.37 | 0.06 | 0.4 |
| mean red blood cell volume | MCV | fL | 92.99 | 5.35 | 2.06 | 92.09 | 5.15 | 2.39 |
| mean hemoglobin volume | MCH | pg | 30.38 | 1.78 | 3.02 | 30.2 | 1.72 | 3.55 |
| mean corpuscular hemoglobin concentration | MCHC | g/L | 325.81 | 12.34 | 0.43 | 327.23 | 12.45 | 0.66 |
| red blood cell distribution width | RDW | % | 13.53 | 1.23 | 4.37 | 13.21 | 1.15 | 3.58 |
| platelet count | PLT | *10^9/L | 175.07 | 66.41 | 1.03 | 188.18 | 71.4 | 1.44 |
| platelet crit | PCT | % | 0.2 | 0.06 | 5.06 | 0.21 | 0.07 | 4.97 |
| mean platelet volume | MPV | fL | 11.19 | 1.34 | 3.9 | 11.17 | 1.35 | 3.46 |
| platelet distribution width | PDW | % | 13.51 | 3.17 | 7.12 | 13.52 | 3.16 | 6.18 |
| hypersensitivity C-reaction Protein | hs-CRP | mg/L | 3.05 | 3.33 | 22.21 | 3.3 | 3.47 | 26.16 |
| Alpha-L-fucosidase | AFU | U/L | 25.91 | 7.49 | 3.3 | 25.85 | 7.65 | 1.73 |
| triglyceride | TG | mmol/L | 1.32 | 0.65 | 6.02 | 1.31 | 0.63 | 5.42 |
| globulin | Gl | g/L | 27.81 | 4.5 | 1.18 | 27.81 | 4.44 | 1.4 |
| glutamyltranspeptidase | GTP | U/L | 27.91 | 15.62 | 10.47 | 26 | 15.64 | 11.89 |
| glutamyl/propanoid ratio | AST/ALT | -- | 1.18 | 0.45 | 3.49 | 1.15 | 0.46 | 2.43 |
| adenosine deaminase | ADA | U/L | 12.76 | 4.23 | 5.12 | 11.3 | 4.4 | 3.27 |
| hemolysis | HA | -- | 3.73 | 2.84 | 5.54 | 3.85 | 2.81 | 5.89 |
| glycosylated hemoglobin A1c | HbA1c |  | 6.17 | 0.79 | 33.12 | 5.78 | 0.69 | 5.53 |
| total bile acids | TBA | µmol/L | 5.27 | 3.39 | 9.51 | 4.5 | 3.22 | 7.77 |
| indirect bilirubin | IBIL | µmol/L | 8.12 | 3.83 | 2.49 | 8.29 | 3.91 | 3.79 |
| albumin/globulin ratio | A/G | -- | 1.37 | 0.27 | 0.26 | 1.36 | 0.27 | 0.29 |
| glucose | GLU | mmol/L | 6.13 | 1.71 | 11.07 | 5.62 | 1.53 | 5.85 |
| sodium | Na | mmol/L | 140.57 | 2.72 | 2.08 | 140.71 | 2.6 | 1.87 |
| phosphorus | P | mmol/L | 1.04 | 0.21 | 2.27 | 1.03 | 0.21 | 1.46 |
| low-density lipoprotein | LDL | mmol/L | 2.12 | 0.9 | 0.78 | 2.53 | 0.88 | 0.55 |
| creatine kinase | CK | U/L | 78.23 | 43.17 | 7.12 | 73.85 | 42.09 | 6.18 |
| total protein | TP | g/L | 65.38 | 7.33 | 1.63 | 65.39 | 7.63 | 4.85 |
| apolipoprotein B | ApoB | g/L | 0.73 | 0.24 | 2.61 | 0.82 | 0.24 | 0.65 |
| calcium | Ca | mmol/L | 2.22 | 0.14 | 0.84 | 2.2 | 0.14 | 0.59 |
| $\alpha$hydroxybutyrate dehydrogenase | $\alpha$-HBDH | U/L | 145.23 | 31.55 | 6.68 | 138.57 | 30.81 | 4.41 |
| creatine kinase isoenzyme | CK-MB | U/L | 18.63 | 6.15 | 6.38 | 17.14 | 5.86 | 5.54 |
| aspartate transaminase | AST | U/L | 21.9 | 7.64 | 7.36 | 21.48 | 7.76 | 9.21 |
| direct bilirubin | DBIL | µmol/L | 3.52 | 1.65 | 4.84 | 3.3 | 1.6 | 6.39 |
| turbidity | Tur | -- | 4.39 | 3.12 | 11.37 | 4.36 | 3 | 8.44 |
| alkaline phosphatase | ALP | U/L | 81.12 | 22.91 | 3.65 | 78.35 | 23.18 | 4.34 |
| lactate dehydrogenase | LDH | U/L | 202.05 | 45.93 | 5.72 | 193.23 | 47.53 | 6.15 |
| lipoprotein/cholesterol | Lp/Ch | -- | 0.87 | 0.07 | 6.7 | 0.89 | 0.07 | 7.78 |
| jaundice | Jaun | -- | 1.11 | 0.45 | 24.14 | 1.15 | 0.47 | 22.32 |
| homocysteine | Hcy | µmol/L | 13.61 | 4.89 | 8.21 | 11.76 | 4.71 | 4.62 |
| apolipoprotein α | Apoα | mg/L | 173.14 | 138.41 | 12.03 | 158.08 | 130.51 | 7.96 |
| apolipoprotein A | ApoA | g/L | 1.31 | 0.3 | 2.3 | 1.31 | 0.33 | 0.72 |
| choline esterase | ChE | U/L | 7004.83 | 2043.44 | 2 | 7197.95 | 2164.3 | 0.28 |
| urea | urea | µmol/L | 5.69 | 1.94 | 7.85 | 4.81 | 1.77 | 4.02 |
| chloride | Cl | mmol/L | 106.68 | 3.65 | 2.27 | 106.83 | 3.48 | 5.39 |
| albumin | ALB | g/L | 37.38 | 5.14 | 0.37 | 37.39 | 5.45 | 0.93 |
| total bilirubin | TBIL | µmol/L | 11.76 | 5.2 | 3.27 | 11.67 | 5.2 | 5.12 |
| glutamic pyruvic transaminase | ALT | U/L | 20.69 | 10.89 | 6.37 | 20.33 | 11.01 | 10.17 |
| serum amylase | AMY | U/L | 74.47 | 28.51 | 4.79 | 71.6 | 27.37 | 3.98 |
| potassium | K | mmol/L | 3.98 | 0.43 | 1.07 | 3.91 | 0.41 | 0.65 |
| magnesium | Mg | mmol/L | 0.84 | 0.09 | 2.6 | 0.84 | 0.08 | 1.56 |
| creatinine | Cr | µmol/L | 75.03 | 20.48 | 9.51 | 66.93 | 18.24 | 4.9 |
| cholesterol | Ch | mmol/L | 3.76 | 1.06 | 0.93 | 4.17 | 1.06 | 0.75 |
| high-density lipoprotein | HDL | mmol/L | 1.12 | 0.31 | 0.77 | 1.15 | 0.34 | 0.85 |
| uric acid | UA | µmol/L | 325.24 | 104.63 | 0.9 | 293.76 | 102.1 | 0.5 |
| max systolic blood pressure | max-SBP | mmHg | 150.1 | 18.76 | 12.14 | 146.93 | 20.09 | 11.92 |
| min systolic blood pressure | min-SBP | mmHg | 110.08 | 13.22 | 12.86 | 109.46 | 13.43 | 12.78 |
| mean systolic blood pressure | mean-SBP | mmHg | 128.85 | 13.64 | 12.12 | 127.06 | 14.85 | 11.89 |
| max diastolic blood pressure | max-DBP | mmHg | 84.25 | 10.33 | 12.27 | 85.34 | 10.87 | 12.21 |
| min diastolic blood pressure | min-DBP | mmHg | 59.64 | 8.02 | 12.69 | 60.95 | 8.27 | 12.32 |
| mean systolic blood pressure | mean-DBP | mmHg | 71.17 | 7.79 | 12.16 | 72.64 | 8.31 | 11.97 |
| max body temperature | max-BT | ℃ | 98.9 | 0.83 | 12.47 | 98.91 | 0.79 | 13.99 |
| min body temperature | min-BT | ℃ | 95.18 | 1.91 | 13.4 | 95.51 | 1.9 | 14.88 |
| mean body temperature | mean-BT | ℃ | 97.18 | 0.92 | 12.51 | 97.39 | 0.96 | 14.01 |
| max respiratory rate | max-RR | times/minute | 37.32 | 0.57 | 10.61 | 37.48 | 0.71 | 10.51 |
| min respiratory rate | min-RR | times/minute | 36.21 | 0.25 | 10.6 | 36.25 | 0.27 | 10.51 |
| mean respiratory rate | mean-RR | times/minute | 36.73 | 0.22 | 10.6 | 36.8 | 0.25 | 10.51 |
| max blood oxygen saturation | max-SpO₂ | % | 19.64 | 1.76 | 12.76 | 20 | 1.52 | 13.11 |
| min blood oxygen saturation | min-SpO₂ | % | 16.78 | 1.32 | 11.48 | 17.27 | 1.42 | 11.5 |
| mean blood oxygen saturation | mean-SpO₂ | % | 18.21 | 1 | 11.23 | 18.65 | 0.9 | 11.38 |
| 1st body temperature | 1st-BT | ℃ | 36.73 | 0.46 | 2.75 | 36.86 | 0.58 | 3.73 |
| 1st heart rate | 1st-HR | bpm | 78.71 | 14.42 | 2.86 | 81.98 | 14.52 | 3.77 |
| 1st respiratory rate | 1st-RR | times/minute | 18.51 | 1.44 | 2.96 | 18.87 | 1.32 | 3.9 |
| 1st systolic blood pressure | 1st-SBP | mmHg | 135.82 | 21.07 | 2.8 | 133.28 | 20.95 | 3.83 |
| 1st diastolic blood pressure | 1st-DBP | mmHg | 74.81 | 11.99 | 2.82 | 76.75 | 11.93 | 3.81 |
| 1st blood oxygen saturation | 1st- SpO₂ | % | 97.17 | 1.83 | 2.91 | 97.42 | 1.74 | 3.86 |
